# Estimating annual prevalence of depression and anxiety disorder in multiple sclerosis using administrative data

**DOI:** 10.1186/s13104-017-2958-1

**Published:** 2017-11-25

**Authors:** Ruth Ann Marrie, Randy Walld, James M. Bolton, Jitender Sareen, John R. Walker, Scott B. Patten, Alexander Singer, Lisa M. Lix, Carol A. Hitchon, Renée El-Gabalawy, Alan Katz, John D. Fisk, Charles N. Bernstein, For the CIHR Team in Defining the Burden and Managing the Effects of Psychiatric Comorbidity in Chronic Immunoinflammatory Disease, Ruth Ann Marrie, James M. Bolton, Jitender Sareen, John R. Walker, Scott B. Patten, Alexander Singer, Lisa M. Lix, Carol A. Hitchon, Renée El-Gabalawy, Alan Katz, John D. Fisk, Charles N. Bernstein, Lesley Graff, Lindsay Berrigan, Ryan Zarychanski, Christine Peschken, James Marriott

**Affiliations:** 1 Department of Internal Medicine, Max Rady College of Medicine, Rady Faculty of Health Sciences, University of Manitoba, Winnipeg, Canada; 2 Department of Community Health Sciences, Max Rady College of Medicine, Rady Faculty of Health Sciences, University of Manitoba, Winnipeg, Canada; 3 Manitoba Centre for Health Policy, Max Rady College of Medicine, Rady Faculty of Health Sciences, University of Manitoba, Winnipeg, Canada; 4 Department of Psychiatry, Max Rady College of Medicine, Rady Faculty of Health Sciences, University of Manitoba, Winnipeg, Canada; 5 Department of Clinical Health Psychology, Max Rady College of Medicine, Rady Faculty of Health Sciences, University of Manitoba, Winnipeg, Canada; 6 Department of Community Health Sciences, Cumming School of Medicine, University of Calgary, Calgary, Canada; 7 Department of Family Medicine, Max Rady College of Medicine, Rady Faculty of Health Sciences, University of Manitoba, Winnipeg, Canada; 8 Department of Anesthesia and Perioperative Medicine, Max Rady College of Medicine, Rady Faculty of Health Sciences, University of Manitoba, Winnipeg, Canada; 9 Departments of Psychiatry, Psychology & Neuroscience, and Medicine, Dalhousie University, Halifax, Canada; 10 Health Sciences Center, GF-543, 820 Sherbrook Street, Winnipeg, MB R3A 1R9 Canada

**Keywords:** Multiple sclerosis, Depression, Anxiety, Epidemiology

## Abstract

**Objective:**

Researchers have developed case definitions to estimate incidence and lifetime prevalence of depression and anxiety disorders in multiple sclerosis (MS) using administrative data. For policymakers however, the prevalence of a disease requiring ongoing treatment during a given period such as annual period prevalence may be more relevant for decision-making. We tested a case definition for annual period prevalence of depression and anxiety disorders in MS using administrative data.

**Results:**

Using population-based administrative (health claims) data from Manitoba, Canada we identified 1922 persons with incident MS from 1989 to 2012, and 11,392 age, sex and geographically-matched controls from the general population. As compared to controls, MS patients had an elevated annual prevalence ratio of depression (1.77; 95% confidence interval [CI] 1.64, 1.91), and anxiety disorders (1.46; 95% CI 1.35, 1.58). The annual prevalence of depression in our matched cohort was similar to that observed in the 2012 Canadian Community Health Survey, although the annual prevalence of anxiety was slightly higher. Administrative data can be used to estimate the annual period prevalence of psychiatric disorders in MS.

**Electronic supplementary material:**

The online version of this article (10.1186/s13104-017-2958-1) contains supplementary material, which is available to authorized users.

## Introduction

The burden of psychiatric illness is high in multiple sclerosis (MS) due to its high prevalence [1], and adverse effects on outcomes such as hospitalizations and mortality [2, 3]. In a systematic review, the prevalence of depression was 23.9% (95% CI 17.4, 30.0%) and of anxiety disorders was 21.9% (95% CI 8.76, 35.0) among those with MS [1]. Previously, we developed case definitions to identify psychiatric comorbidity in the MS population using administrative (health claims) data [4], and estimated the incidence and lifetime prevalence of psychiatric comorbidity. However, when planning health services, the prevalence of a disease requiring ongoing treatment during a given period such as a year (i.e., annual period prevalence) is more useful than lifetime prevalence [5–7]. While health system changes may not reduce the lifetime prevalence of disease, annual prevalence could be reduced if effective treatments were provided and recurrences prevented [8, 9]. Herein, we built on prior work [4] and tested an approach to measure annual period prevalence of depression and anxiety disorders in MS using administrative data.

## Main text

### Setting

This retrospective matched cohort study used administrative databases for Manitoba, Canada, held in the Manitoba Population Data Repository at the Manitoba Centre for Health Policy. Health care is publicly funded in Manitoba and, these databases prospectively capture health services for > 98% of the population. We used the Population Registry (which provided dates of birth, death and health care coverage, sex, and region of residence [postal code]); the Discharge Abstract Database (which provided hospitalizations, including admission and discharge dates, and up to 25 diagnoses recorded using International Classification of Disease [ICD]-9/10 codes); Medical Services (physician claims, which provided service date and single ICD-9-CM physician-coded diagnosis); and Drug Program Information Network (DPIN, which provided all community-dispensed prescriptions including drug name, dispensation date, and drug identification number [DIN]). DPIN became available in 1995, but all other databases were available from April 1, 1984-March 31, 2012. We linked these databases deterministically at the individual level using a de-identified unique personal health identification number.

### Study populations

We applied a validated case definition to identify Manitoba residents with MS during the study period [10]. We classified individuals as having MS if they had ≥ 3 hospital or physician or prescription claims for MS. Hospital or physician claims for MS were identified using ICD-9/10 codes of 340/G35. Prescription claims for MS were identified using DINs for MS-specific disease-modifying therapies. We limited the analysis to incident cases of MS by removing cases with any demyelinating disease claims within 5 years before the index date. Given that the databases were available as of 1984, the earliest index date for an incident case was 1989. Next, a cohort matched 5:1 on sex, year of birth ± 5 years, and forward sortation area (i.e. first 3 digits of postal code) was generated. As this was part of a larger study including other immune-mediated diseases [10], anyone with claims for demyelinating disease, inflammatory bowel disease, rheumatoid arthritis and related disorders were excluded. Each control was assigned the index date of its matched case.

### Psychiatric disorders

We applied validated case definitions (Additional file 1: Table S1) to identify individuals who ever met definitions for depression or anxiety disorders (any anxiety disorder, not limited to generalized anxiety disorder) [4]. Since the case definitions for depression and anxiety disorder included prescription claims, we included a binary covariate in our analyses indicating whether the disorder occurred before or after prescription data became available in 1995. We estimated lifetime prevalence by assuming that if an individual met the case definition for depression or an anxiety disorder, he or she remained affected thereafter, as long as he or she was still living in Manitoba. Our previous case definition validations used self-reported lifetime diagnoses of depression or anxiety disorders, and disorders ever recorded as present on medical records review as reference standards [4].

To estimate annual prevalence, once a person met the case definition, he or she was counted as an annual prevalent case if there were ≥ 2 physician claims or one hospital claim for the psychiatric disorder in that year; for hospital claims the disorder had to be the most responsible diagnosis. Therefore an individual who stopped receiving care in a given year did not need to meet the full case definition to be counted as a case once care resumed. We did not consider prescription claims in the annual prevalence definition because of the frequent off-label use of antidepressants and anxiolytics in the MS population for symptom management [11]. To test these “annual” case definitions, we compared the annual prevalence estimates in the matched population to those reported for the general population in the Canadian Community Health Survey-Mental Health (CCHS-MH) [12]. In 2012, the CCHS-MH data (N = 25,113) estimated the annual prevalence of depression and generalized anxiety disorders using the World Health Organization (WHO) *Composite International Diagnostic Interview* (*CIDI*), regardless of whether these conditions were diagnosed and treated.

### Covariates

Covariates included sex (male as reference group), age (18–24 [reference group], 25–44, 45–64, ≥ 65), socioeconomic status (SES) in quintiles (worst quintile as reference group), and region (urban or rural [reference group]). We linked participants’ postal codes to dissemination-area level census data to determine SES as defined by the Socioeconomic Factor Index version 2 (SEFI-2). The SEFI-2 incorporates data regarding average household income, percent of single parent households, unemployment rate and high school education rate; scores < 0 indicate better SES [13]. Winnipeg (population > 700,000) and Brandon (population > 47,000) were designated as urban regions. We included year in the regression models (below) to evaluate temporal trends.

### Analysis

The prevalence estimates were age and sex-standardized to the 2010 Canadian population, and 95% confidence intervals (95% CI) were determined using a negative binomial distribution. We report age-specific prevalence estimates using age groups 18–24, 25–44, 45–64, ≥ 65 years, to be consistent with the CCHS-MH. We compared the annual prevalence estimates between the MS and matched cohorts using negative binomial regression models (to account for overdispersion) adjusting for covariates (above), for which we report prevalence ratios (PR) and 95% CIs. We included the natural logarithm of person-years as the model offset to account for variable follow-up duration, and used generalized estimating equations with an exchangeable correlation structure to account for dependence of repeated prevalence estimates within individuals. In separate models, we tested if the average annual rate of change differed between the two cohorts by including an interaction term (cohort*year).

We performed statistical analyses using SAS V9.4 (SAS Institute Inc., Cary, NC).

### Results

We identified 3514 incident cases of MS and 17,526 matched individuals without MS. The two cohorts were well-matched at the index date (Table 1).

**Table 1:** Characteristics of incident multiple sclerosis (MS) and matched cohorts at the index date

Characteristic	MS matches (n = 17,526)	MS (n = 3514)
Female, n (%)	12,697 (72.4)	2544 (72.4)
Age at diagnosis, mean (SD)	40.8 (12.5)	40.8 (12.5)
Duration of follow-up from the index date (years), median (IQR)	10.5 (5.0, 16.3)	10.3 (4.90, 16.1)
Region of residence, n (%)		
Urban	11,685 (66.7)	2344 (66.7)
Rural	5841 (33.3)	1170 (33.3)
Socioeconomic Factor Index Score^a^	− 0.18 (0.87)	− 0.24 (0.89)

^a^Values less than zero indicate higher socioeconomic status

In 2011, the crude lifetime prevalence of depression was 44.8% (95% CI 42.9, 46.6%) in the MS cohort and 27.1% (95% CI 26.3, 27.8%) in the matched cohort. The crude annual prevalence of depression in the MS cohort was 12.6% (95% CI 11.5, 13.9%) and in the matched cohort was 7.7% (95% CI 7.2, 8.1%). The age and sex-standardized annual prevalence of depression was 10.7% (95% CI 9.02, 12.6%) in the MS cohort, 64% higher (PR 1.64; 95% CI 1.35, 1.98) than in the matched cohort (6.5%; 95% CI 5.9, 7.2%).

The annual prevalence of depression was lower than the lifetime prevalence of depression at all ages (Table 2). After accounting for differences in the sex distribution of our matched population and the Canadian general population, the annual prevalence of depression in the Canadian general population, as drawn from the CCHS-MH, was similar to that observed in our matched cohort in all groups except those aged ≥ 65 years (Table 2).

**Table 2:** Age-specific prevalence (95% confidence interval) of depression and anxiety disorders per 100 population in 2011

Annual					Prevalence ratio MS: matches	Lifetime		Prevalence ratio MS: matches
Age group	MS	Matches	CCHS-MH^a^	CCHS-MH reweighted^b^		MS	Matches	
Depression								
18–24	12.1 (7.3, 16.8)	8.3 (6.5, 10.1)	7.1^c^ (5.9, 8.3)	8.0^c^ (5.9, 10.1)	1.45^d^ (0.93, 2.27)	37.2 (28.9, 45.5)	23.6 (20.6, 26.6)	1.58^f^ (1.22, 2.04)
25–44	13.0 (11.3, 14.8)	8.1 (7.5, 8.7)	5.4 (4.6, 6.3)	6.1 (4.3, 7.9)	1.61^e^ (1.38, 1.87)	45.7 (42.5, 48.9)	27.9 (26.8, 29.0)	1.64^e^ (1.51, 1.77)
45–64	11.7 (9.4, 14.0)	6.7 (5.9, 7.4)	4.5 (3.9, 5.1)	5.0 (3.5, 6.5)	1.76^e^ (1.40, 2.22)	45.4 (40.8, 50.0)	26.5 (24.9, 28.0)	1.72^e^ (1.53, 1.93)
≥ 65	s	6.5 (3.9, 9.0)	1.6 (1.2, 2.1)	1.7 (0.6, 2.8)	1.45^d^ (0.93, 2.27)	36.8 (21.1, 52.6)	24.0 (19.1, 28.9)	1.53^g^ (0.95, 2.46)
Anxiety disorder								
18–24	5.8 (2.5, 9.1)	7.6 (5.9, 9.3)	2.4 (1.7, 3.1)	3.1 (1.4, 4.8)	0.76^h^ (0.41, 1.40)	43.0 (34.1, 51.9)	33.6 (30.0, 37.2)	1.28^l^ (1.01, 1.62)
25–44	6.3 (5.1, 7.5)	5.4 (4.9, 5.9)	2.9 (2.4, 3.4)	3.1 (1.7, 4.5)	1.18^i^ (0.96, 1.46)	50.0 (46.6, 53.4)	37.9 (36.6, 39.2)	1.32^e^ (1.22, 1.42)
45–64	6.1 (4.4, 7.8)	4.7 (4.0, 5.3)	3.0 (2.5, 3.5)	3.4 (2.0, 4.8)	1.31^j^ (0.96, 1.79)	48.4 (43.6, 53.1)	39.0 (37.2, 40.9)	1.24^e^ (1.11, 1.38)
≥ 65	s	3.1 (1.3, 4.9)	1.2 (0.8, 1.6)	1.2 (0.1, 2.3)	0.57^k^ (0.07, 4.35)	47.4 (29.5, 65.2)	38.5 (32.3, 44.7)	1.23^m^ (0.82, 1.85)

^a^Canadian Community Health Survey-Mental Health (CCHS-MH) estimates; ^b^ CCHS-MH estimates re-weighted to match male–female proportion in the matched study population; ^c^ CCHS-MH age group is 15–24 years; *s* suppressed due to privacy and confidentiality considerations; ^d^ p = 0.10; ^e^ p < 0.0001; ^f^ p = 0.0005; ^g^ p = 0.077; ^h^ p = 0.38; ^i^ p = 0.12; ^j^ p = 0.088; ^k^ p = 0.58; ^l^ p = 0.038; ^m^ p = 0.32

**Fig. 1:**
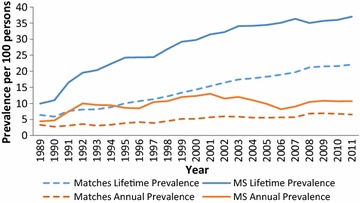
Lifetime and annual prevalence of depression in the multiple sclerosis (MS) and matched population

After adjustment for age, sex, SES, region of residence and year, the annual prevalence of depression (IRR 1.76; 95% CI 1.62, 1.90) was higher in the MS cohort than the matched cohort over the entire study period (Additional file 1: Table S1). Female sex, older age (versus persons aged 18–24 years), urban versus rural residence, and lower SES were associated with an increased prevalence of depression. The lifetime and annual prevalence increased slightly over time, but less in the MS than the matched cohort (both p < 0.0001, Fig. 1).

In 2011, the crude lifetime prevalence of anxiety was 48.9% (95% CI 47.1, 50.8%) in the MS cohort and 37.9% (95% CI 37.1, 38.7%) in the matched cohort. The crude annual prevalence of anxiety disorders in the MS cohort was 6.2% (95% CI 5.3, 7.1%) and in the matched cohort was 5.3% (95% CI 5.7, 6.0%). The age- and sex-standardized annual prevalence of anxiety disorders in the MS cohort was 5.6% (95% CI 4.6, 6.9%), 13% higher (1.13; 95% CI 0.90, 1.42) than in the matched cohort (5.0%; 95% CI 4.4, 5.6%).

Annual prevalence was lower than lifetime prevalence for anxiety disorders in all age groups (Table 2). After accounting for differences in the sex distribution of our matched population and the Canadian general population, the annual prevalence of anxiety in the Canadian general population, as drawn from the CCHS-MH, was slightly lower than that observed in our matched cohort in all groups except those aged ≥ 65 years (Table 2).

After adjustment for age, sex, SES, region of residence and year, the annual prevalence of anxiety disorder (PR 1.46; 95% CI 1.35, 1.58) was higher in the MS cohort than the matched cohort over the entire study period (Additional file 1: Table S1). Female sex, older age (as compared to persons aged 18-24 years), urban versus rural residence, and lower SES were associated with an increased prevalence of anxiety. The lifetime and annual prevalence increased slightly over time, but less in the MS than the matched cohort (both p < 0.0001).

### Discussion

In this population-based study the lifetime and annual prevalence of depression and anxiety disorders were higher in the MS population than in a matched general population cohort without MS. In the MS population, the annual period prevalence of depression and anxiety disorders changed minimally over time, suggesting consistent patterns of health care utilization for those conditions over time. Accounting for the higher proportion of women in our sample than in the CCHS-MH sample, age-specific annual prevalence estimates of depression in our matched cohort fell within the bounds of the CCHS-MH estimates for all age groups except age ≥ 65 years. Our age-specific anxiety disorder estimates were slightly higher than those reported in the CCHS-MH, which may reflect the fact that the CCHS-MH identified only generalized anxiety disorder, while our case definition captured anxiety disorders more generally. Overall, the findings suggest that our proposed approach to identifying annual period prevalence is appropriate and could be applied to other conditions. Comparable work is limited. The Canadian Chronic Disease Surveillance System identifies annual prevalence of mental illness on the basis of any hospitalization or physician visit for mental illness but this case definition includes dementia and developmental disorders, and does not attempt to distinguish diagnoses due to feasibility concerns [1, 2]. Other investigators have developed administrative case definitions for depression or anxiety, but have not explicitly distinguished annual and lifetime prevalence [3, 4].

Several demographic factors were associated with the prevalence of depression and anxiety. Women had an increased prevalence of both disorders, consistent with prior studies in MS [14]. As reported in the general population, urban residence was associated with an increased prevalence of depression [15]. Lower SES was associated with an increased prevalence of depression and anxiety, consistent with prior studies showing that lower annual household income is associated with an increased lifetime prevalence of depression in MS [16], and that persons with MS with lower levels of education had greater depressive symptoms [17].

## Limitations

Administrative data may be subject to diagnostic misclassification bias. However, we employed a validated case definition for MS, and the case definitions used to identify lifetime depression and anxiety disorder were developed and validated in two MS populations [4, 18]. We did not validate our case definitions for annual prevalence of depression or anxiety disorders at the individual level, but rather used population-level comparisons. Nonetheless, we identified the expected demographic relationships with the psychiatric disorders studied. Study strengths include the large study population, and use of population-based data sources and concurrent controls.

Administrative data can be used to estimate annual period prevalence of psychiatric disorders. The prevalence of depression and anxiety disorders is higher in the MS cohort than in an age-, sex- and geographically-matched cohort without MS.

## Additional file

**Additional file 1: Table S1.** Adjusted rate ratios and 95% confidence intervals for the association between multiple sclerosis (MS) and prevalence of depression and anxiety disorder. **Figure S1.** Lifetime and annual prevalence of anxiety in the multiple sclerosis (MS) and matched populations.
