# Supplementary material for: Estimating annual prevalence of depression and anxiety disorder in multiple sclerosis using administrative data
Source: BMC Res Notes. 2017 Nov 25;10:619. doi: 10.1186/s13104-017-2958-1 (PMC5702192; doi:10.1186/s13104-017-2958-1)
Supplement: Supplementary file 1 — Additional file 1: Table S1. Adjusted rate ratios and 95% confidence intervals for the association between multiple sclerosis (MS) and prevalence of depression and anxiety disorder. Figure S1. Lifetime and annual prevalence of anxiety in the multiple sclerosis (MS) and matched populations. [file 13104_2017_2958_MOESM1_ESM.docx]

Figure S1. Lifetime and annual prevalence of anxiety in the multiple sclerosis (MS) and matched populations

Table S1. Adjusted^a^ rate ratios and 95% confidence intervals for the association between multiple sclerosis (MS) and prevalence of depression and anxiety disorder

| **Variable** | **Depression** | **P-value** | **Anxiety Disorder** | | **P-value** | |
| --- | --- | --- | --- | --- | --- | --- |
| *Cohort* |  |  |  |  | |  |
| Matches | 1.0 | <0.0001 | 1.0 | <0.0001 | |  |
| MS | **1.76**  **(1.62, 1.90)** |  | **1.46**  **(1.35, 1.58)** |  | |  |
| *Sex* |  |  |  |  | |  |
| Male | 1.0 | <0.0001 | **1.0** | <0.0001 | |  |
| Female | **1.67**  **(1.53, 1.83)** |  | **1.32**  **(1.22, 1.43)** |  | |  |
| *Age (yrs)* |  |  |  |  | |  |
| 18-24 | 1.0 |  | 1.0 |  | |  |
| 25-44 | **1.54**  **(1.21, 1.97)** | 0.0006 | 0.98  (0.82, 1.16) | 0.79 | |  |
| 45-64 | **1.48**  **(1.15, 1.89)** | 0.0021 | 0.85  (0.72, 1.01) | 0.064 | |  |
| ≥65 | 1.07  (0.82, 1.41) | 0.62 | 0.72  (0.58, 0.89) | 0.0026 | |  |
| *Socioeconomic status* |  |  |  |  | |  |
| Quintile 1  (lowest) | **1.28**  **(1.14, 1.44)** | <0.0001 | **1.34**  **(1.21, 1.48)** | <0.0001 | |  |
| Quintile 2 | **1.18**  **(1.05, 1.32)** | 0.0041 | **1.17**  **(1.07, 1.27)** | 0.0008 | |  |
| Quintile 3 | **1.12**  **(1.02, 1.24)** | 0.025 | **1.11**  **(1.00, 1.23)** | 0.045 | |  |
| Quintile 4 | **1.14**  **(1.03, 1.26)** | 0.012 | **1.21**  **(1.10, 1.33)** | <0.0001 | |  |
| Quintile 5  (highest) | 1.0 |  | 1.0 |  | |  |
| *Region* |  |  |  |  | |  |
| Rural | 1.0 | <0.0001 | 1.0 | <0.0001 | |  |
| Urban | **1.36**  **(1.24, 1.48)** |  | **1.39**  **(1.28, 1.51)** |  | |  |
| *Year^b^* | **1.023^c^**  **(1.016, 1.029)** | <0.0001 | **1.014^d^**  **(1.007, 1.021)** | 0.0001 | |  |

1. Models for depression, and anxiety disorders also include term to adjust for whether prescription claims were used in the case definition; b-Year refers to average annual change; c- year effect in cases PR 1.005; 95%CI: 0.996-1.014, year effect in controls PR 1.030; 95%CI: 1.023-1.037; d- year effect in cases PR 0.991; 95%CI: 0.982-1.001, year effect in controls PR 1.021; 95%CI: 1.014-1.029
